# The Applications of Promoter-gene-Engineered Biosensors

**DOI:** 10.3390/s18092823

**Published:** 2018-08-27

**Authors:** Yingzhu Feng, Zhangzhang Xie, Xuanlong Jiang, Zhen Li, Yuping Shen, Bochu Wang, Jianzhong Liu

**Affiliations:** 1 School of Life Sciences, Sun Yat-Sen University, Guangzhou 510275, China; xiezhzh9@mail.sysu.edu.cn (Z.X.); jiangxuanlong_17@163.com (X.J.); Lizzi@163.com (Z.L.); yupp_123@163.com (Y.S.); 2 Key Laboratory of Bio-Theological Science and Technology of Ministry of Education, College Bioengineering, Chongqing University, Chongqing 400030, China; wangbc2000@126.com

**Keywords:** biosensor, promoter, sensitivity, specificity, high-throughput screening (HTS), genetic promoter chip, “push and pull” mode, toxicity

## Abstract

A promoter is a small region of a DNA sequence that responds to various transcription factors, which initiates a particular gene expression. The promoter-engineered biosensor can activate or repress gene expression through a transcription factor recognizing specific molecules, such as polyamine, sugars, lactams, amino acids, organic acids, or a redox molecule; however, there are few reported applications of promoter-enhanced biosensors. This review paper highlights the strategies of construction of promoter gene-engineered biosensors with human and bacteria genetic promoter arrays with regard to high-throughput screening (HTS) molecular drugs, the study of the membrane protein’s localization and nucleocytoplasmic shuttling mechanism of regulating factors, enzyme activity, detection of the toxicity of intermediate chemicals, and probing bacteria density to improve value-added product titer. These biosensors’ sensitivity and specificity can be further improved by the proposed approaches of Mn^2+^ and Mg^2+^ added random error-prone PCR that is a technique used to generate randomized genomic libraries and site-directed mutagenesis approach, which is applied for the construction of bacteria’s “mutant library”. This is expected to establish a flexible HTS platform (biosensor array) to large-scale screen transcription factor-acting drugs, reduce the toxicity of intermediate compounds, and construct a gene-dynamic regulatory system in “push and pull” mode, in order to effectively regulate the valuable medicinal product production. These proposed novel promoter-engineered biosensors aiding in synthetic genetic circuit construction will maximize the efficiency of the bio-synthesis of medicinal compounds, which will greatly promote the development of microbial metabolic engineering and biomedical science.

## 1. Introduction

With the development of life sciences and DNA molecular technology, we can discover the key gene, new enzyme, or protein that controls the main signaling pathway for synthesis of a desired product. Many genes or enzymes are directly regulated by a transcription factor without effector binding; however, some genes not only need a transcription factor, but also compound effector binding for dynamically controlling gene expression [1,2]. Access to large of quantities of these dynamic regulatory components is critical for the discovery of new biosensors for novel applications [3,4,5]. Therefore, promoters, transcription factors and molecular effectors collectively contribute to the discovery of biosensors and significantly draw our attention (Table 1).

### 1.1. Strategies for the Construction of Promoter-Enhanced Biosensor

Because promoters are an important component for initiating gene expression, the strong promoters can be identified through comparative transcriptional analysis, which has a high gene expression ratio. The gene expression profile tells us that for up- or down-regulated genes, which strong or weak promoters can be in response to specific molecules, the strong promoter can be identified through microarray-based transcriptional analysis [6,7,8].

#### 1.1.1. Synthesizing Promoters to Increase Their Properties

**Figure 1:**
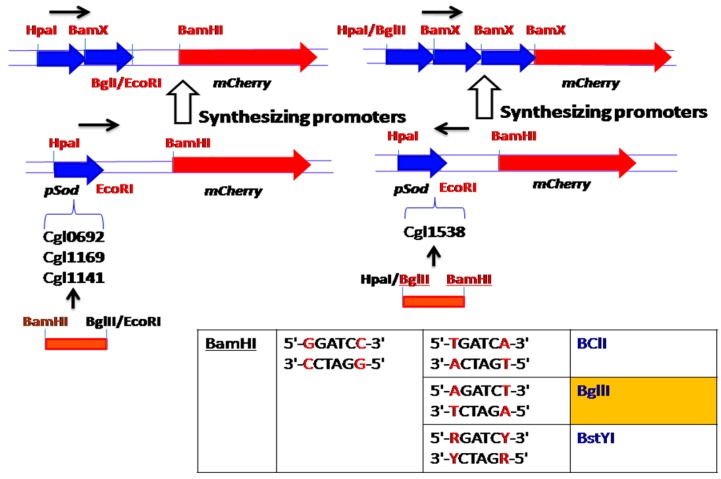
Synthesizing promoters of *Corynebacterium glutamicum* by DNA brick assembly technique.

Strong promoters can be selected according to comparative transcriptional analysis, and their strength can be characterized by fusing an immunofluorescent protein, such as the *mCherry* or *GFP* gene, for fluorescence testing, which enables HTS of transcription factor-acting molecular drugs. However, most of the time, the activity of a single promoter in response to a compound is so weak and insufficient that it cannot be effectively characterized; as a result, promoters need to be synthesized to enhance their performance through certain DNA assemble techniques, like the DNA brick method, which utilizes isocaudarner ligation for connecting a series of promoters [9,10]. The approach of synthesizing promoters of *Corynebacterium glutamicum* from two directions are illustrated in Figure 1.

#### 1.1.2. Approaches of Mn^2+^ and Mg^2+^ Added Random, Error-Prone PCR and Site-Directed Mutagenesis for Further Improving a Synthesized Promoter’s Performance

**Figure 2:**
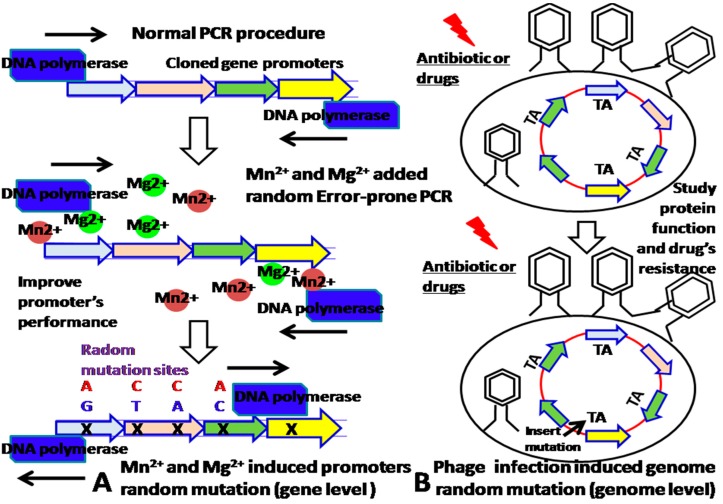
(**A**). Mn^2+^ and Mg^2+^ added random, error-prone PCR approach introduces the gene mutation sites at the gene level. (**B**) Phage infection introduces random genome mutation sites.

The improvement of promoter’s properties is beneficial for construction of sensitive biosensors. Although we can increase the strength of promoters by synthesizing them, how to further improve their sensitivity and specificity are needed our more consideration. Given that metal Mn^2+^ and Mg^2+^ added random, error-prone PCR random mutagenesis throughout the entire promoter’s sequence can add random mutated sites into the promoter PCR fragments, which leads to a change in their performance, the mutants can be screened by fluorescence-activated cell sorting (FACS) analysis at various drug concentrations [11]. In addition, the mutation sites can be identified by sequence analysis and validated by the site-directed mutagenesis approach. Furthermore, site-directed mutagenesis approach could determine which mutation site contributed to the specificity of promoter. These newly introduced mutation sites are the promoter’s specific binding sites of the transcription factor–molecular effector complex (Figure 2A) at the gene level. If the bacteria is infected by a phage, the packaging phage can cause random mutations to be inserted into the TA sites of the bacterial gene’s promoter [11,12] (Figure 2B).

## 2. Applications of Promoter Gene-Engineered Biosensors

### 2.1. Construction of High-Throughput Screening Platform for Screening Transcription Factor-Acting Molecules and Anti-Tumor Drugs with High Pharmacological Activity

**Figure 3:**
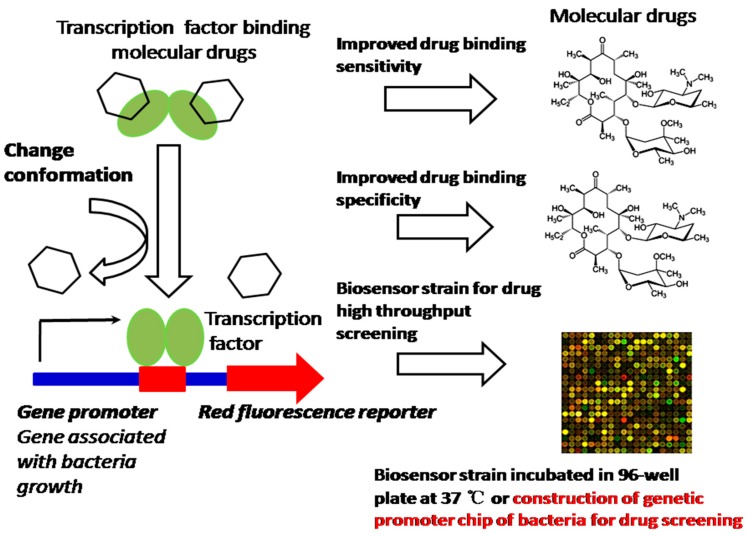
Construction of biosensor strain and genetic promoter chip of bacteria for high-throughput screening (HTS) transcription factor (TF)-acting molecular drugs.

**Figure 4:**
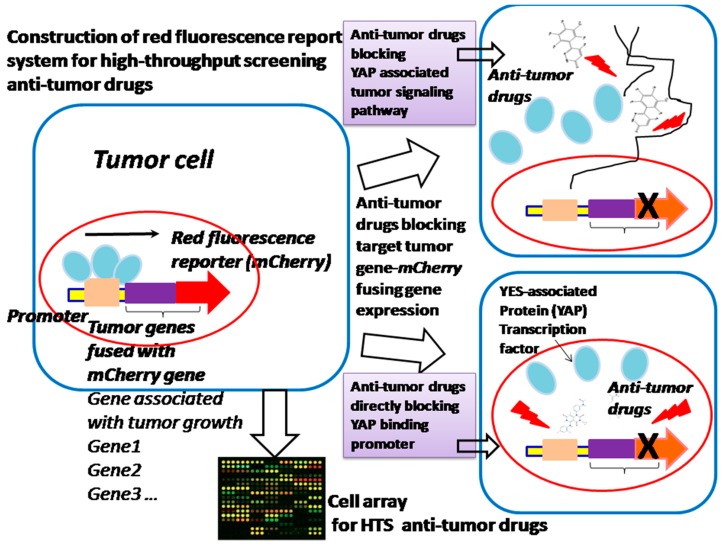
Construction of HTS platform (cell array) for HTS anti-tumor drugs. Anti-tumor drugs block YES-associated protein (YAP) transcription of tumor genes, which can be applied for drug HTS.

In many cases, bacteria trigger signaling cascades through transcription factors, as a response to a specific compound for coping with the environment. These transcription factors also play an important role in bacteria’s physiological activities, such as the regulation of salt and cell envelope stress that counteract extracytoplasmic stresses, NaCl and hormone balance, acting as a metal transporter protecting against oxidative stress, copper homeostasis, counteracting hydrogen peroxide-mediated oxidative stress, and gene expression inhibited by nitric oxide (NO) [13]. All these exhibited activities of bacteria due to transcription factors are highly effective devices, sensitively and specifically bind small compounds and trigger allosteric responses to control the transcription of one or more genes. Because they sense various molecular effectors, transcription factors have been engineered to enable us to regulate valuable product production, including dicarboxylic acids, alcohols, phenylpropanoids, lactone etc. [14]. Nevertheless, the promoter still exhibits a binding saturation effect and the gene expression is not really sufficient, which results in insufficiency of the drug screening. The strategy of synthesizing promoters, which will promote more transcription factor (TF)–molecular effectors specifically binding gene promoters, will increase the DNA translation efficiency, thus leading to the improvement of drug screening. Therefore, promoter-enhanced biosensors and the construction of a genetic promoter chip of bacteria with synthesizing promoters could enable us to construct an HTS platform to screen TF-acting compounds; for example, we have screened up-regulated genes (ratio ≥ 6.0) of mutated *Corynebacterium glutamicum* (ER6937R42) that are correlated with high production of l-Ornithine, as well as up-regulated genes (ratio ≥ 7.0) of mutated *Corynebacterium glutamicum* (16-17-CPVF-ALE) that are correlated with high production of putrescine [14,15]. The corresponding promoters can be found, assembled, and fused with *mCherry* or *EGFP* genes for the construction of an HTS platform for detecting the drugs l-Ornithine and putrescine [15] (Figure 3). We can further improve the sensitivity and specificity of these biosensors by site-directed mutagenesis approaches, to increase the sensitivity of detection of l-Ornithine and putrescine in bacteria. The method proposed can be extended to screening of other TF-acting molecular drugs or anti-tumor compound screening by cell array (Figure 4), especially those drugs associated with the growth and energy metabolism of bacteria [16] (Figure 3) or tumor growth and invasion [17,18] (Figure 4).

### 2.2. Performance-Enhanced Promoter Can Be Applied for Studying the Membrane Protein’s Localization and Enzyme Activity

**Figure 5:**
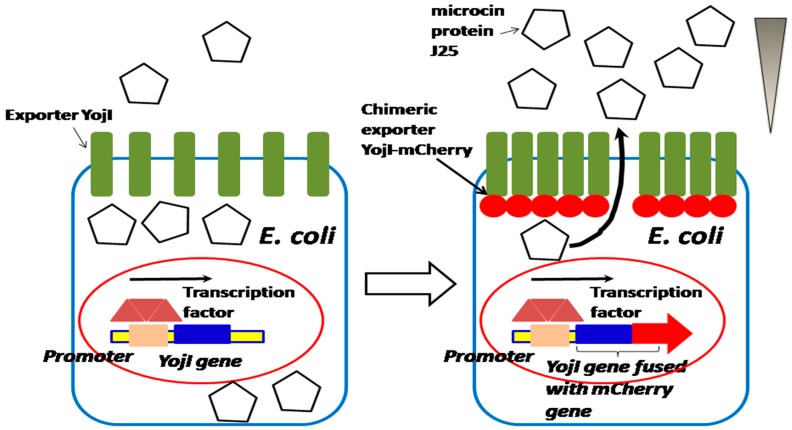
A performance-enhanced promoter is applied for the construction of a chimeric exporter, for the purpose of studying the activity of *YojI*–*mCherry* during the export of microcin protein J25.

**Figure 6:**
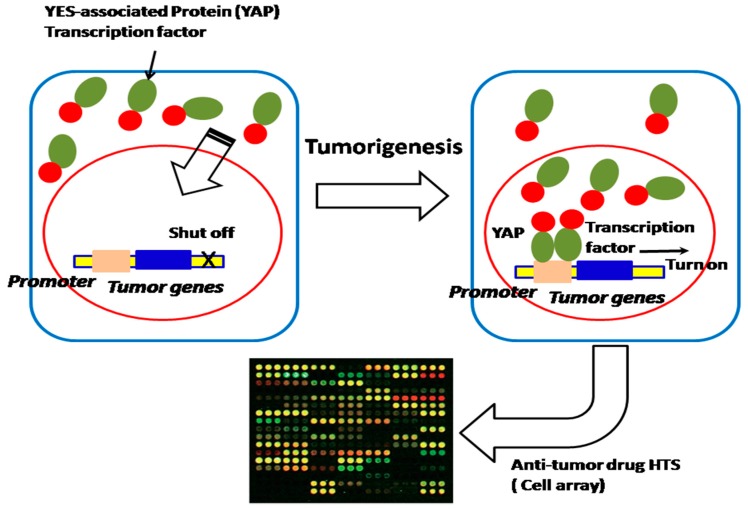
A performance-enhanced promoter is applied for the construction of an HTS platform for HTS of anti-tumor drugs and studying nucleocytoplasmic shuttling regulating factor YAP’s nucleocytoplasmic localization and tumorigenesis, which controls tumor growth and invasion.

**Figure 7:**
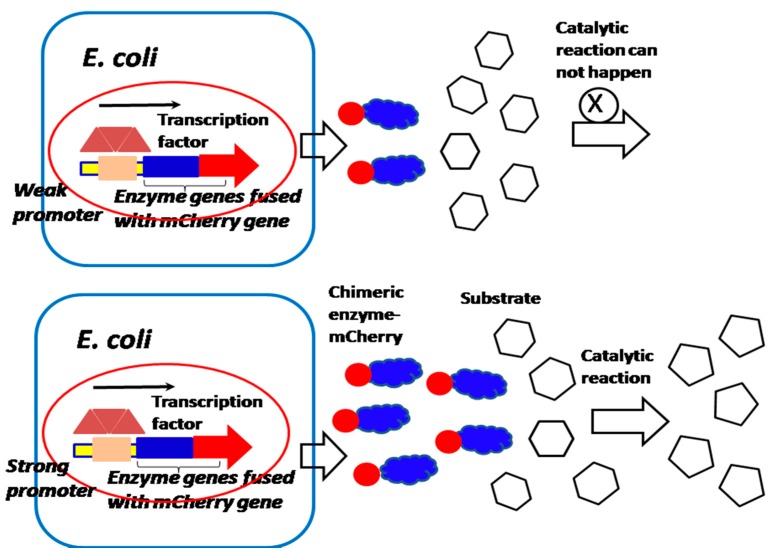
A performance-enhanced promoter is applied for studying the enzyme activity. High concentration of the enzyme will produce high catalytical activity, if the enzyme is not saturated by a substrate.

Because the quantity of most membrane proteins of bacteria on the cell surface is limited [19], the performance-enhanced promoter can be cloned before the *mCherry* or *EGFP* gene fused with the membrane protein gene, in order to increase the chimeric protein expression for studying membrane proteins’ localization and activity (Figure 5). Interestingly, if the *mCherry* gene and *EGFP* gene are fused with nucleocytoplasmic shuttling regulating factor YES, which is associated with the transcription factor (YAP) that controls tumor growth and invasion, the YAP nucleocytoplasmic behavior of movement and localization can be observed and controlled [17,18]. Therefore, the investigation of the nucleocytoplasmic shuttling mechanism of YAP enables us to deeply understand the tumorigenesis and oncology mechanism, and find more effective tumor therapy (Figure 6). Furthermore, if both *mCherry* and *EGFP* gene are fused with individual interactive proteins or enzymes, we can investigate the protein–protein interactions and bacteria–host cell interactions associated with the pathogenesis of infectious diseases and the mechanism of the catalytic reaction of enzymes and their activity in the cell array [20,21,22,23] (Figure 5 and Figure 7). 

### 2.3. Utilization of a Promoter for Construction of a Sensitive Biosensor for Probing the Density of Bacteria

**Figure 8:**
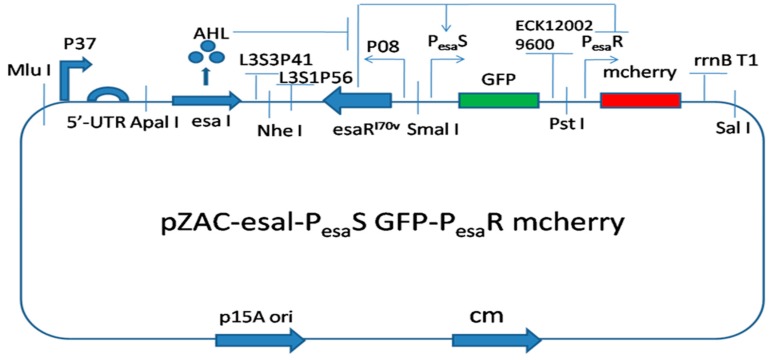
Both red and green fluorescent protein (*mCherry* and *GFP* genes) probe the density of bacteria. The promoter of the *GFP* gene (PesaS) respnods to low density of bacteria. The promoter of *mCherry* gene (PesaR) responds to high density of bacteria.

**Figure 9:**
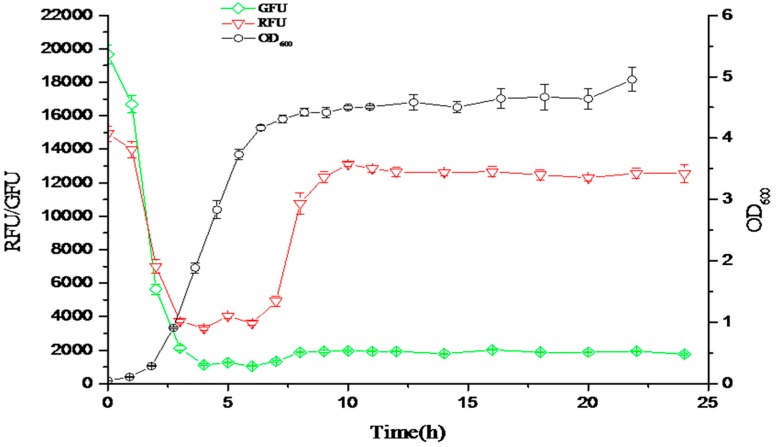
The responsive curves of the fluorescent ratio (OD_535_/OD_600_) in response to the density of bacteria. The green curve is the green fluorescent ratio (OD_535_/OD_600_) responding to the low density of bacteria. The red curve is the red fluorescent ratio (OD_535_/OD_600_) responding to the high density of bacteria. The black curve is the cell density of bacteria (OD_600_).

Due to the strong luminescent properties of immunofluorescent proteins, we could integrate both low and high bacteria density-responsive promoters into the genetic regulatory circuit for probing the cell density [24,25] (Figure 8). This biosensor could be in response to *N*-Acyl-homoserine lactone (AHL), which is the signal molecule of the quorum sensing system of bacteria and probes the growth density of bacteria, which in turn provides instructions for how to balance cell growth density, in order to improve the valuable product production (Figure 9).

### 2.4. Mn^2+^ and Mg^2+^ Added Random Error-Prone PCR Mutagenesis Approach for the Construction of the Biosensor “Library” That Is Regulated by Inhibitory Proteins

**Figure 10:**
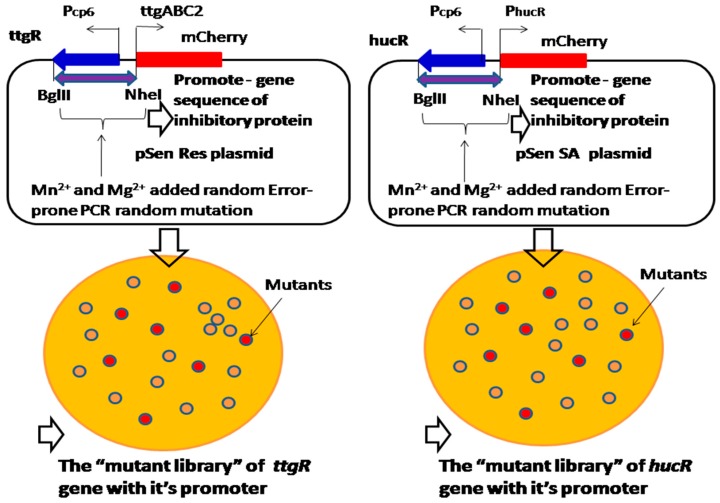
Mn^2+^ and Mg^2+^ added random error-prone PCR mutagenesis approach for randomly mutating the entire inhibitory protein gene with its promoter sequence, in order to construct the “mutant library” to screen the sensitivity of the biosensors.

Resveratrol and shikimic acid synthesis is inhibited by ttgR and hucR inhibitory proteins, which respond to resveratrol and shikimic acid [26,27]. Most of the time, the expression of ttgR or hucR constitutive genes is repressed by an inhibitory protein without binding resveratrol or shikimic acid; however, the gene expression will be initiated under the condition of the resveratrol or shikimic acid binding, which triggers the allosteric response that de-repress the inhibitory effect [26]. Because the Mn^2+^ and Mg^2+^ added random error-prone PCR mutagenesis approach can introduce random mutation sites into the DNA sequence, the properties of the gene with the promoter of inhibitory proteins can be improved for resveratrol and shikimic acid binding, and the strength of the mutated gene with the promoter could be screened from the “mutant library” (Figure 10). Therefore, random mutation of a promoter by Mn^2+^ and Mg^2+^ added error-prone PCR can increase a promoter’s sensitivity to the inhibitory protein, which rapidly de-represses the inhibitory effect and promotes resveratrol and shikimic acid production.

### 2.5. Integration of Both a Promoter-Based Biosensor and a Bacterium–Host Cell Interactive Mechanism for High-Throughput Screening of Meningitis Drugs

**Figure 11:**
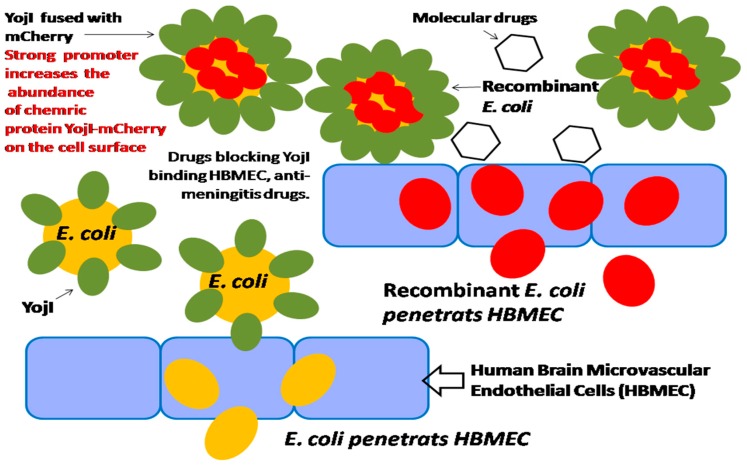
Integration of a promoter-based biosensor and bacterium–host cell interactive mechanism for HTS of meningitis drugs.

In our previous studies, we found that in the membrane protein of *E. coli*, YojI can mediate the interaction of *E. coli* and human brain microvascular endothelial cells (HBMECs), by using both the human and *E. coli* proteome chips in conjunction with cell labeling techniques for the discovery of microbial and host factors. We identified that YojI binds to the interferon-alpha receptor (IFNAR2) on the surface of HBMECs and mediates *E. coli* adhesion to the host cells, and is an important virulence factor for *E. coli* invasion of HBMECs [28]. It is reported that YojI is also a chemically-induced biosensor for regulating its own expression, a leucine-responsive regulatory protein; Lrp controls the expression of YojI, which regulates exporting of toxin J25 [28,29,30]. Therefore, the strong promoter can be identified and cloned before the chimeric *YojI*–*mCherry* gene to highly initiate the gene expression, and to strongly incorporate the YojI–mCherry chimeric protein onto the cell surface. When the recombinant *E. coli* infects HBMECs, the strong promoter initiates the chimeric YojI–mCherry protein expression will amplify the invasion signaling of *E. coli* in HBMEC. As a result, the strength of the promoter of *YojI* gene is correlated with the invasion efficiency of *E. coli*. The bacterium–host cell interactive mechanism, integrated with high-performance promoter, can be effectively utilized for HTS of meningitis drugs (Figure 11).

### 2.6. Construction of Promoter-Engineered Genetic Circuit for the Detection of Intermediate Toxic Compounds to Improve the Synthesis of Value-Added Medicinal Product Yields

**Figure 12:**
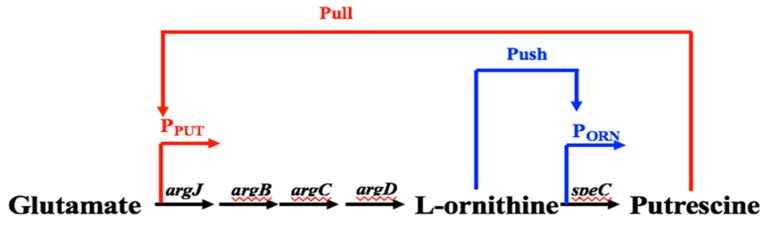
Construction of dynamic genetic circuit in “push” and “pull” mode to promote conversion of l-ornithine to putrescine.

The promoters for regulating pathway expression in response to intermediates can create a link between a cell’s metabolic state and expression of the metabolic pathway. It is reported that the coupling of synthetic promoters and metabolic engineering can improve the production of valuable chemicals. We have identified native promoters that respond to l-Ornithine and putrescine in our previous studies. On the one hand, once the l-Ornithine binds the transcription factors, it will trigger the allosteric response to activate the expression of l-ornithine carboxylase, which facilitates the rapid synthesis of l-ornithine and the conversion of l-ornithine to putrescine. This is the “push” regulatory mode of l-Ornithine that promotes the synthesis of putrescine. On the other hand, excessive accumulation of putrescine will bind its promoter to activate the expression of *N*-acetyl glutamic acid synthetase system ArgCJBD [31,32]. ArgCJBD will promote the conversion of glutamic acid to synthesis of l-ornithine and supplement the consumption of l-ornithine, which increases the intracellular level of l-ornithine, and further promote the transformation to putrescine [15,31]. This is the remote “pull” regulatory mode for the synthesis of putrescine. We expect to integrate both “push” and “pull” biosensors into an integrated genetic circuit, in order to dynamically regulate the synthesis of putrescine in *Corynebacterium glutamicum*. This “push” and “pull” regulatory mode will continuously promote the synthesis of l-ornithine and conversion of l-ornithine to putrescine, as the ArgCJBD route can supplement the consumption of l-ornithine, which pushes forward the conversion of l-ornithine to putrescine (Figure 12). With the above-mentioned approaches, we could synthesize the promoters in order to increase their responsive strength, and thus increase the product production and apply the above-mentioned Mn^2+^ and Mg^2+^ added random error-prone PCR mutagenesis approach, to further enhance the promoter’s performance and greatly improve the synthesis of putrescine. 

## 3. Discussion and Conclusions

In this paper, a range of approaches have been proposed for engineering promoter-gene components for the construction of sensitive biosensors. Because the fluorescence intensity of *mCherry* and *EGFP* protein expression are correlated with the promoter’s strength, the promoter-gene component can be engineered for the construction of biosensors with human and bacteria arrays for diverse applications, such as HTS of transcription factor-acting and anti-tumor drugs, increasing the abundance of membrane proteins and enzymes to study their activities, observing of movement and localization of nucleocytoplasmic shuttling factors for tumorigenesis and oncology mechanism investigation, applying both mCherry and EGFP proteins for probing the density of bacteria to improve synthesis of high valuable product, and constructing a dynamic genetic circuit for monitoring the intermediate toxic compound, which can be applied in food and environmentally toxic substance testing. Interestingly, the YojI regulatory biosensor combined with the *E. coli* invasion mechanism of HBMEC can be utilized for HTS of meningitis drugs and the discovery of other infection disease therapy [33,34,35]. Although there are many applications of biosensors proposed in this paper for human health and the environment, the properties of the promoter-gene component, including the sensitivity and specificity of the biosensor, are not too hard to handle, because the Mn^2+^ and Mg^2+^ added random error-prone PCR mutagenesis approach could introduce random mutation sites to the promoter-gene component and a corresponding fragment to construct the “mutant library”; the mutants, with improved properties, can be screened by this “library”. The promoter strength can be further improved by easily synthesizing the promoter to increase the responsive strength of biosensors. As this proposed method develops, the new enzyme activity with a designed biosensor can be discovered. This novel gene switch allow us to stringently control virulent protein expression and study more new enzyme functions [26,31].

In conclusion, the promoter-gene component engineered strategy has the potential to revolutionize recent biotechnological development and allow the rapid engineering of required circuits, in order to increase the particular value-added product production in the bio-medicinal field.

## Figures and Tables

**Table 1:** Promoters, transcription factors, and molecular effectors engineered for the construction of biosensors.

Engineered Component	Approaches	Reference
Engineered molecular effectors	(1) Fluorescence of strain HF19 harboring P_BAD_-*gfpuv* reporter plasmid (Ppcc442) and expressing AraC-mev (Ppcc423-mev), in the presence of the indicated concentration of small molecule inducers (“effectors”), such as mevalonate, succinic acid, l-arabinose, Triacetic acid lactone. (2) MphR inducers are macrolides, such as erythromycin, oleandomycin, nabomycin, pikromycin, methymycin, josamycin.	[4,5]
Engineered transcription factor	l-arabinose-responsive transcription factor engineered to specifically respond to the level of d-arabinose, acid lactone, and mevalonate.	[5]
Engineered promoter	An oleic acid biosensor replacing the native FadR-regulated *fadBA* promoter with a synthetic two copies of promoter into the strong phage *T7* pomoter.	[5,9,10]
